# Bad Choices Make Good Stories: The Impaired Decision-Making Process and Skin Conductance Response in Subjects With Smartphone Addiction

**DOI:** 10.3389/fpsyt.2019.00073

**Published:** 2019-02-22

**Authors:** Julia Machado Khoury, Luiz Filipe Silva Codorino Couto, Douglas de Almeida Santos, Vitor Hugo de Oliveira e Silva, João Pedro Sousa Drumond, Letícia Lopes de Carvalho e Silva, Leandro Malloy-Diniz, Maicon Rodrigues Albuquerque, Maila de Castro Lourenço das Neves, Frederico Duarte Garcia

**Affiliations:** ^1^Department of Mental Health, Universidade Federal de Minas Gerais–UFMG (Federal University of Minas Gerais), Belo Horizonte, Brazil; ^2^Department of Clinical Medicine, Faculty of Health and Human Ecology, Belo Horizonte, Brazil; ^3^Post-Graduation Program in Molecular Medicine (Pós-Graduação em Medicina Molecular), School of Medicine, Universidade Federal de Minas Gerais–UFMG (Federal University of Minas Gerais), Belo Horizonte, Brazil; ^4^Department of Sports, Universidade Federal de Minas Gerais–UFMG (Federal University of Minas Gerais), Belo Horizonte, Brazil; ^5^INCT of Molecular Medicine, Universidade Federal de Minas Gerais–UFMG (Federal University of Minas Gerais), Belo Horizonte, Brazil; ^6^Unité Inserm U1073, Rouen, France

**Keywords:** decision-making, game of dice task, Iowa gambling test, skin conductance, smartphone addiction, somatic markers

## Abstract

**Introduction:** Smartphone Addiction (SA) has caused negative consequences and functional impairments in college students, such as reduction of academic performance and impairment in sleep quality. Studies have shown that individuals with chemical and behavioral dependencies have a bias in decision-making process, which leads to short-term advantageous choices even if they cause long-term harm. This bias in decision-making process is accompanied by a change in somatic markers and is associated with the development and maintenance of addictive behavior. The decision-making process and the measurement of physiological parameters have not yet been analyzed in SA. The neuropsychological and physiological characterization of the SA can contribute to its approach with the other dependency syndromes and to its recognition as a disease.

**Objective:** we aimed to evaluate the decision-making process under risk and under ambiguity in individuals with SA and to measure the physiological parameters that accompany this process.

**Method:** We compared the performance in the Iowa Gambling Task (IGT), Game of Dice Task (GDT) and skin conductance response (SCR) between 50 individuals with SA and 50 controls.

**Results:** Smartphone dependents presented a profile of impairment in decision-making under ambiguity, without impairment in decision-making under risk. They demonstrated lower SCR before disadvantageous choices, higher SCR after rewards and lower SCR after punishments during decision-making, which suggests difficulty in recognizing disadvantageous alternatives, high sensitivity to rewards, and low sensitivity to punishments.

**Conclusion:** The impairment in the decision-making process in smartphone dependents is similar to that found in other chemical and behavioral addictions, such as alcohol addiction, gambling disorders and pathological buy. The impairment in decision under ambiguity with preservation of decision under risk may reflect dysfunction of implicit emotional processes without dysfunction of explicit cognitive process. This profile can contribute to the recognition of SA as a behavioral dependence and to guide specific preventive and therapeutic strategies.

## Introduction

Smartphone Addiction (SA) is a new construct that has been associated with morbidity, such as reduction in academic and labor performances (1–9), sleep disorders (10, 11), impairments in interpersonal relationships (12–14), and an increased risk for in traffic accidents (12–14). The screening prevalence of SA ranges from 25% in the USA (15), 27% in Hong Kong (16), and 38% in Spain (17), to 43% in Brazil (18).

The key features of SA are the interruption or reduction of important social, occupational, or recreational activities due to smartphone use (19–24); constant preoccupations with the possibility of device absence (13, 21, 24, 25); an increased frequency and intensity of use despite negative consequences (19–21, 24); a difficulty in controlling use (12, 19, 23, 25–27); and the presence of dysphoric symptoms when the contact with the smartphone is precluded (17, 19–21, 24, 25, 27, 28). SA has not been included in the diagnostic and statistical manuals of mental disorders (DSM) (29) despite the large range of data already available.

Similar to what occur in other dependence syndromes (30–37), decision making may be impaired in SA. This impairment in decision making process may predispose subjects to become addict to smartphones and increase the negative consequences of SA. For example, preferring smartphone use, as a short-term reward, may be a risk factor for the development of addiction, and may result in long-term impairments such as reduction of interpersonal relationships quality.

Decision making can be divided in two subtypes, accordingly to probability of its results: (1) decision making under ambiguity; and (2) decision making under risk (38). In decision-making under ambiguity, the consequences of decision and probability of outcomes are implicit in the decision. The subject must initially infer about the quality of the options available by the memory processing of previous decisions (cognitive feedback and emotional feedback). Decision-making under ambiguity reflects more strongly the reality of daily decisions, which are made without prior certainty of the probability of each outcome (39). In decision making under risk, the subject receives explicit rules and can choose through the risk calculation of the options before making any choice. Decision making under risk is more frequently observed in situations where there is certainty of the probability of each outcome (40). Previous studies have demonstrated impairment in performance in decision-making in behavioral addictions (33, 41–44). Most studies that investigated the two types of decision making concomitantly in these patients demonstrated impairment in decision making under ambiguity, with preservation of decision-making under risk, as suggested in studies with pathological buyers (44) and pathological gamblers (45, 46). We present, in Supplemental Material, a compilation of studies that have evaluated the decision-making process in individuals with behavioral dependencies.

Physiological parameters and somatic markers contribute to decision making process (47). Skin conductance response (SCR), is one of the physiological parameters modified during decision making process (47). Accordingly to somatic marker hypothesis, the modification of SCR after losses and gains reflects the appraisal of decision outcome and the sensibility to punishments and rewards (47). Anticipatory SCR, in face of a decision process, is a somatic marker, that may be interpreted as warning signal, helping the subject to choose advantageous alternatives and to avoid disadvantageous alternatives in future decisions (47). In real life, the increase of SCR before disadvantageous choices and after losses during decision-making help to postpone rewards and to guide choices based on long term benefits, helping achieve goals and objectives. Subjects with addictions present a decreased SCR when confronted with disadvantageous choices and after losses. They also present an increased SCR after wins on decision-making tests (31, 35, 48). These findings suggest that subjects with addictions present impairment in recognizing disadvantageous alternatives, hypersensitivity to rewards and hyposensitivity to punishments. These pattern of decision making process contribute to the onset and maintenance of addictive disorders (49, 50).

Three studies assessed decision making under risk in subjects with SA. They used the “Intertemporal Choice Test” (ICT) to access decision under risk and do not measured physiological parameters (51–53). As far as we know, until know, no study has evaluated decision-making under ambiguity in SA. However, the study of the two types of decision making is important so that we can compare the neuropsychological profile of individuals with SA with individuals with other dependency syndromes. Individuals with SA are more likely to present a decision-making profile similar to that presented by the majority of individuals with other behavioral dependencies (preservation of the decision under risk with impairment of the decision under ambiguity) and slightly different from that presented by the majority of individuals with chemical dependencies (impairment in the two types of decision-making). If this is true, it will be another fact that suggests the inclusion of SA within the behavioral dependency syndromes, thus increasing the validity of this new construct.

Furthermore, no studies have measured alterations in SCR during decision making process in subjects presenting SA, which makes it difficult to interpret the impairments found in the performances in the tests that measure decision making.

We hypothesize that smartphone dependents make more disadvantageous decisions on tests that measure decision-making under ambiguity and have no impairments in decision-making under risk when compared to control subjects. Moreover, we hypothesize that higher scores on the screening scale for SA are correlated with worse performances in both decision-making under ambiguity test. Finally, we expect that smartphone dependents present a lower anticipatory SCR before disadvantageous choices compared to SCR before advantageous choices; and higher SCR after wins compared to SCR after losses during both decision-making tests. Therefore, in this study we assessed decision-making under risk and under ambiguity in subjects at risk for SA and measured alterations in SCR that accompanied this process.

## Materials and Methods

### Participants and Sample

For this study we randomly selected 100 graduate students aged between 18 and 25 years, from the sample of our previous study for the validation of the Brazilian version of the “Smartphone Addiction Inventory (SPAI-BR)” (18). For this study we divided the selected subjects in two groups of 50 subjects each, accordingly to the SPAI-BR results. We included, in the positive screening group, subjects presenting positive screening for SA; and not presenting other psychiatric disorders accordingly to Brazilian version of the “Mini-International Neuropsychiatric Interview” (MINI). In the control group we included subjects with negative screening for SA; and not presenting other psychiatric disorders accordingly to MINI. We excluded subjects presenting past history of traumatic brain injury; current or past history of neurological disorder; current or past history of psychiatric disorder/substance use disorder; current use of psychiatric and/or neurological drugs; an intelligence quotient inferior to 80.

### Instruments

- Brazilian Smartphone Addiction Inventory (SPAI-BR): the SPAI is a screening scale for SA, which was constructed and validated in Taiwan in 2014 (54). It has 26 items and four factors: “compulsive behavior,” “functional impairment,” “withdrawal syndrome,” and “tolerance.” In 2017, we translated and validated the SPAI for use in Brazil (18). The SPAI-BR is positive for SA screening if there is at least seven positive answers (18). We chose the SPAI-BR as the measure for SA because no established diagnostic criteria for the construct currently exist. Also, SPAI-BR has good psychometric characteristics, and it is the only scale for SA screening that has been validated for use in Brazil.
- Raven Progressive Matrices Test–General Scale: it assess non-verbal intellectual performance of all age individuals (55). The test checks a person's ability to grasp meaningless figures, establish relationships between them, make inference about the nature of the figure that would complete the system of implicit relationships, and develop a systematic method of reasoning. The test consists of 60 items, each successful one scores at one point. The total score is transformed into intelligence quotient (IQ) by a mathematical formula. The instrument was validated for use in Brazil in 2003 (56).
- Game of Dice Task (GDT): In this study, we used a computerized version of GDT, which was developed by Brand et al. (57) and validated for Brazilian use by Rzezak et al. (58). In GDT the rules are explicit and stable for gains and losses as well as for the probabilities of winning throughout the test. Individuals are required to predict the outcomes of a dice throw. They must decide between different alternatives (a single number or a combination of numbers) that are explicitly related to a specific amount of gain and loss and which have obvious probabilities of an advantageous result (1: 6–4: 6). Because rules for profit and loss are explicitly provided, individuals can calculate the risk associated with each alternative from the beginning of the test and can use strategies to maximize profit. Each choice is related to a specific gain and loss that depends on the probability of occurrence of the choice (one number: U$1,000 loss/gain; combination of two numbers: U$500 loss/gain; combination of three numbers: U$200 loss/combination of four numbers: U$100 loss/gain). Participants are also informed that they are expected to make a total of 18 throws. To analyze decision-making under risk, authors ranked the choices of one or two numbers as risky or disadvantageous, and the choices of three or four numbers as non-risky or advantageous. A total score is calculated by the sum of advantageous choices (three and four numbers) minus the sum of disadvantageous choices (one and two numbers). Therefore, a positive overall score indicates a better test performance and a lower propensity to make risky choices.
- Iowa Gambling Task (IGT): In this study, we used a computerized version of IGT, which was developed by Bechara et al. (59) based on the original test (60) and validated for Brazilian use by Malloy-Diniz et al. (61). In the computerized version of IGT, participants see four decks of cards on the computer screen (A, B, C, and D) and must choose one card at a time, which can generate a gain or a gain followed by a loss. After choosing 100 cards, the test ends automatically. For each 10 cards from the deck “A,” the participant earns U$ 1,000, but there are 5 unpredictable losses of U$250, causing a total loss of U$250. For each 10 cards from the deck “B,” the participant earns U$1,000, but there is also a large loss of U$1,250, causing a total loss of U$250. On the other hand, for every 10 cards in decks C and D, the patients earn only U$500, but the losses are also smaller (ranging from U$25 to U$75 on deck C and U$250 on deck D), leading to a total gain of U$250. In sum, mounts A and B are equivalent in terms of total loss and mounts C and D are equivalent in terms of total gain. In long term, decks A and B are disadvantageous and decks C and D are advantageous.
- Skin Conductance Response (SCR): SCR was measured using the NeXus4® physiological data meter and obtained by applying a 0.5 V voltage current to electrodes positioned on the index and middle fingers of each participant's non-dominant hand. SCR was measured in Micro-Siemens (μS) and the fluctuation of SCR was considered significant if >0.1 μS (62, 63). The following SCR measures were carried out: post-rewards SCR (measured after money wins), post-punishment SCR (measured after money losses), advantageous anticipatory SCR (measured before advantageous choices), and disadvantageous anticipatory SCR (measured before disadvantageous choices). In accordance to the recommendations of Nikolaidou et al. (37), the time window for the measurement of SCR in rewards and punishments began in the 2nd s after the result of the choice until the 5th s. For the anticipatory SCR, the measurement began in the 6th s after the result of the previous choice until the 9th s. SCR was quantified dividing the area under the SCR curve by the time determined in seconds (μS/s). The interval between the choices in both tests was set at 10 s to ensure non-overlap between SCRs and to allow skin conductance to return to the baseline level between the choices.

### Procedures

Participants were submitted to a self-filled questionnaire composed of the following parts: (1) sociodemographic questions (i.e., biological gender; self-declared race/skin color; date of birth; marital status; monthly family income); (2) questions about health conditions (current or past history of cranioencephalic trauma; current or past history of neurological disorder; current or past history of psychiatric disorder; current use of psychiatric and/or neurological medication; current use of licit or illicit drugs); (3) Raven Progressive Matrix Test; and 4-SPAI-BR. After completing the questionnaires, participants performed the GDT and the IGT, concomitantly with the measurement of the SCR. Procedures were performed under the supervision of a psychiatrist and two trained students.

### Statistical Analysis

The sample size was calculated using G Power 3.1.0 software. The parameters used were: *F*-test, two-tailed, hypothetical effect size of 0.29 [according to the study of Tang et al. for the assessment of decision-making under risk in smartphone dependents (53)], *p* < 0.05, and test power of 0.80. For these parameters, the sample size should be of 80 individuals (40 in each group). Considering a possible loss of 20%, 100 individuals were recruited to participate in the study (50 for the case group and 50 for the control group).

The results were submitted to statistical analysis using the statistical package SPSS version 23 (IBM Corporation, Rochester, MN), and “*p*” was set at < 0.05 as significance level. The normality of the data was analyzed by the Kolmogorov- Smirnov test.

For descriptive analyses, means, standard deviations, medians, quartiles, and intervals for the continuous variables were calculated. For the categorical variables, absolute, and relative frequencies and proportions were calculated. As the data had a non-normal distribution, we used the Mann-Whitney test to compare means of independent samples and the Wicoxon test to compare means of dependent samples. The Spearman Correlation Coefficient was used to calculate the correlation between SPAI-BR, IGT, and GDT scores. Effect sizes were calculated as suggested by Field (64).

## Results

At the endpoint, 10 subjects were excluded from the sample: four individuals interrupted the questionnaire during the procedure, and six failed to measure SCR. Of the 90 individuals composing the final sample, 47 (52.22%) were female and 43 (47.77%) were male. In addition, 47 (52.22%) individuals were smartphone dependents and 43 (47.77%) were controls. Regarding sex, there were 25 (27.77%) women and 22 (24.44%) men in the SA group; and 22 (24.44%) women and 21 (23.33%) men in the control group. The mean age was 22.39 (±1.67) years. The average monthly family income was U$ 1,762 (±712). The mean IQ was 115.89 (±6.18). The sociodemographic characteristics of the sample can be seen in Table 1.

**Table 1:** Sociodemographic characteristics of the sample.

**Characteristics**		***N***	**%**
Gender	Female	47	52.2
	Male	43	47.8
Marital status	Married	5	5.6
	Not-married	85	94.4
Race/skin color	White	55	61.1
	Not-white	35	38.9
IQ range	Average average	15	16.7
	High average	47	52.2
	Superior	27	30
	High superior	1	1.1
Monthly family income	Up to $814	5	5.6
	From U$814 to U$1,628	13	14.4
	From U$1,628 to U$2,442	8	8.9
	From U$2,442 to U$3,257	8	8.9
	From U$3,257 to U$4,071	8	8.9
	From U$4,071 to U$5,428	5	5.6
	Above U$5,428	25	27.8
	Do not know/did not answer	18	20

### Decision Making Process Under Ambiguity and Under Risk in SA and Controls

**Figure 1:**
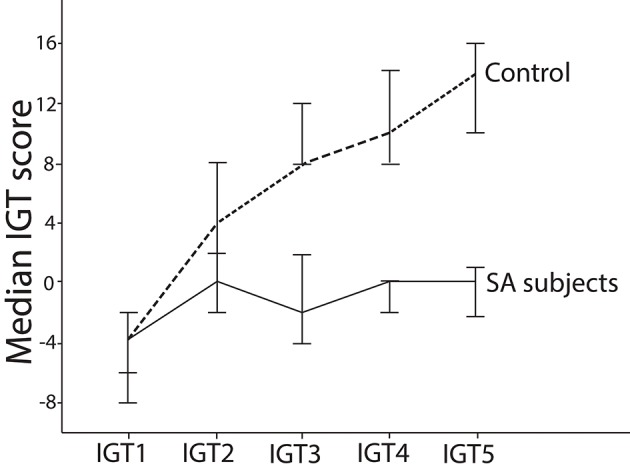
Median score per block in IGT in individuals of the smartphone dependents group and control group. This figure shows that smartphone dependents score lower than controls in the IGT as they progress through the test. This figure also shows that only in Block 1 the control subjects had negative scores (more disadvantageous than advantageous choices), while in blocks 2, 3, 4, and 5 they had positive scores (more advantageous than disadvantageous choices). On the other hand, smartphone dependents do not show tendency of overall increase on scores throughout the test. In addition, in all blocks the scores were negative (i.e., more disadvantageous than advantageous choices) or equal to zero (the same amount of advantageous and disadvantageous choices). Throughout all the game, the scores of the control group were higher than the scores of the smartphone dependents group.

The variables presented a non-normal distribution in the Kolmogorov-Smirnov test (*p* < 0.001). The control group presented higher total score in the IGT, with a large effect size, when compared to the smartphone dependents group (*z* = –6.094, *p* < 0.001, ES = 0.64). The score in Block 1 of the IGT did not differ between the two groups (*z* = –0.057, *p* = 0.955). The score in Block 2 was higher, with an average effect size, in the control group when compared to the smartphone dependents group (*z* = –3.308, *p* = 0.001, ES = 0.35). The scores in Blocks 3, 4, and 5 were higher, with a large effect size, in the control group when compared to the smartphone dependents group (*z* = –5.250, *p* < 0.001, ES = 0.55; *z* = –5.216, *p* < 0.001, ES = 0.55; and *z* = –5.381, *p* < 0.001, TE = 0.57, respectively). Figure 1 shows the median scores by blocks on the IGT in the control group and in the smartphone dependents group.

There was no significant difference in the GDT score between the smartphone dependents group and the control group (*z* = –0.831; *p* = 0.416). This data can be seen in Table 2.

**Table 2:** Comparison between the smartphone dependents and the controls with respect to the medians of the scores in the total IGT, IGT by blocks, and GDT.

	**Smartphone dependents**		**Controls**		***Z***	***p***	**ES**
	**M**	**IQ**	**M**	**IQ**			
IGT total score	−6	22	30	22	−6.094	< 0.001*	0.64
IGT block 1 score	−4	6	−4	8	−0.057	0.955	NA
IGT block 2 score	0	8	4	10	−3.308	0.001*	0.35
IGT block 3 score	−2	10	8	10	−5.250	< 0.001*	0.55
IGT block 4 score	0	8	10	10	−5.216	< 0.001*	0.55
IGT block 5 score	0	8	14	10	−5.381	< 0.001*	0.57
GDT	12	12	14	10	−0.831	0.416	NA

ES, effect size; IQ, interquartil interval; M, median.

* p < 0.05; NA, not applicable.

### Correlations

As shown in Table 3, we found a positive weak correlation between the IGT score and the GDT score (rho = 0.288, *p* = 0.006). In addition, we found negative and moderate correlation between the IGT score and the SPAI-BR score (rho = −0.516, *p* < 0.001). No significant correlations were found between the GDT score and the SPAI-BR score (rho = −0.056; *p* = 0.602).

**Table 3:** Spearman coefficient correlation between variables.

	**SPAI-BR**	**IGT**
**SPAI-BR**		
IGT	−0.516*	
GDT	−0.056	0.288*

* p < 0.05.

### Skin Conductance

The skin conductance values presented a non-normal distribution in the Kolmogorov-Smirnov test (*p* < 0.05). Therefore, the Wilcoxon test was performed for the paired skin conductance analysis.

In the control group, we found a higher SCR anticipatory to the disadvantageous choices in relation to the advantageous choices in the IGT (*z* = –4.069; *p* < 0.001; ES = 0.62). On the other hand, the smartphone dependents group presented a higher SCR anticipatory to the advantageous choices in relation to the disadvantageous choices in the IGT (*z* = –5.037; *p* < 0.001; ES = 0.73) (Table 4).

**Table 4:** Comparison between SCR anticipatory to advantageous choices and SCR anticipatory to disadvantageous choices in the smartphone dependents group and in control group during IGT performance.

	**SCR anticipatory to advantageous choices**		**SCR anticipatory to disadvantageous choices**		***Z***	***p***	**ES**
	**Median**	**IQ**	**Median**	**IQ**			
SD	1.1122	1.2656	1.0709	1.0053	−5.037	< 0.001*	0.73
Controls	0.7667	0.9220	0.8582	0.9876	−4.069	< 0.001*	0.62

ES, effect size; IQ, interquartil interval; SD, smartphone dependents.

* p < 0.05.

In the control group, there was a higher SCR after punishments compared to the SCR after rewards in the IGT (*z* = –3.595, *p* < 0.001, ES = 0.55). However, in the smartphone dependents group, there was a higher SCR after rewards compared to SCR after punishments in the IGT (*z* = –3.810, *p* < 0.001, ES = 0.56). These data can be visualized in Table 5.

**Table 5:** Comparison between SCR after rewards and SCR after punishments in the smartphone dependents group and in control group during IGT performance.

	**SCR after rewards**		**SCR after punishments**		***Z***	***p***	**ES**
	**Median**	**IQ**	**Median**	**IQ**			
SD	1.1326	1.0969	1.0072	0.9953	−3.810	< 0.001*	0.56
Controls	0.8173	1.1218	0.8622	1.1758	−3.595	< 0.001*	0.55

ES, effect size; IQ, interquartil interval; SD, smartphone dependents.

* p < 0.05.

In the control group, there was a higher SCR anticipatory to disadvantageous choices in relation to the SCR anticipatory to advantageous choices in the GDT (*z* = –3.968; *p* < 0.001; ES = 0.61). However, in smartphone dependents group, there was a higher SCR anticipatory to advantageous choices in relation to the SCR anticipatory to disadvantageous choices in the GDT (*z* = –4.996; *p* < 0.001; ES = 0.73).These data can be visualized in Table 6.

**Table 6:** Comparison between SCR anticipatory to advantageous choices and SCR anticipatory to disadvantageous choices in the smartphone dependents group and in control group during GDT performance.

	**SCR anticipatory to advantageous choices**		**SCR anticipatory to disadvantageous choices**		***Z***	***p***	**ES**
	**Median**	**IQ**	**Median**	**IQ**			
SD	0.6185	0.6179	0.4388	0.7342	−4.996	< 0.001*	0.73
Controls	0.3369	0.7786	0.5143	0.6337	−3.968	< 0.001*	0.61

ES, effect size; IQ, interquartil interval; SD, smartphone dependents.

* p < 0.05.

In the control group, there was a higher SCR after punishments compared to the SCR after rewards in the GDT (*z* = –3.212, *p* = 0.001, ES = 0.49). However, in the smartphone dependents group, there was a higher SCR after rewards in relation to the SCR after punishments (*z* = –4.318, *p* < 0.001, ES = 0.63). These data can be seen in Table 7.

**Table 7:** Comparison between SCR after rewards and SCR after punishments in the smartphone dependents group and in control group during GDT performance.

	**SCR after rewards**		**SCR after punishments**		***Z***	***p***	**ES**
	**Median**	**IQ**	**Median**	**IQ**			
SD	0.5533	0.7072	0.4567	0.6505	−4.318	< 0.001*	0.63
Controls	0.4633	0.6478	0.5267	0.6830	−3.212	0.001*	0.49

ES, effect size; IQ, interquartil interval; SD, smartphone dependents.

* *p < 0.05*.

## Discussion

According to our initial hypothesis, smartphone dependents presented impairment in the decision-making under ambiguity with preservation of decision-making under risk. Our sample presented a negative correlation between the severity of SA in the SPAI-BR questionnaire and the performance in decision making under ambiguity in the IGT. Regarding the physiological parameters, smartphone dependents presented, in both decision-making tests, a decrease in SCR before disadvantageous choices, an increase in SCR after rewards, and a decrease in SCR after punishments, which also corroborated our initial hypothesis. To the best of our knowledge, this is the first study investigating decision-making performance under risk and ambiguity in smartphone dependents both in behavioral and physiological levels.

Except for the performance in the first block of the IGT, smartphone dependents presented a worse performance in all IGT blocks and total IGT when compared to controls. These findings strongly suggest that smartphone dependents chose more disadvantageous alternatives than advantageous alternatives during almost the whole test. We suggest that smartphone dependents present an impaired decision-making under ambiguity. When the rules of the game are not explicit and the outcomes are uncertain, smartphone dependents presented a difficulty making advantageous long-term choices. They tend to prefer advantageous alternatives in the short term, even when they bring greater future losses. Their decision-making pattern is in parallel to the physiological modifications found in smartphone dependents during decision-making under ambiguity. The lower anticipatory SCR before disadvantageous choices indicates a possible deficit in generating somatic markers that usually work as warning signals against disadvantageous decisions (47). Consequently, it is possible that smartphone dependents have deficits in transferring their emotional reactions to create anticipatory warning signals that would guide future decisions (48, 65). Moreover, the increase in SCR after rewards, associated with the decrease in SCR after punishments, suggests that smartphone dependents appraise decision outcome differently from control subjects. Smartphone dependents presented a greater sensitivity to rewards and a lower sensitivity to punishments. Their decisions are guided preferably by the search for rewards rather than the avoidance of punishments. Therefore, when an alternative generates high reward, it is chosen even if it generates greater punishment in long term.

The same profile of decision-making under ambiguity in smartphone dependents has been described in subjects presenting substance and gambling addictions (33, 41, 66–69), suggesting that SA is part of the addictive syndromes. This “myopia for the future” profile at decisive moments can contribute to the initiation and maintenance of addictive behaviors, as individuals perform the behavior because they are more sensitive to the immediate reward caused by it and less sensitivity to the damage they can cause in various areas of life (47). Therefore, the greater sensitivity to “likes” and “comments” on social networks for example may be a vulnerability factor for the development of SA and for the maintenance of dysfunctional use of smartphones, even when there are losses or possibility of damages in several areas of life, such as reduction of academic and work performance and impairments in interpersonal relationships. This can contribute to the reduction of functionality and to the generation of suffering to self and / or others, essential characteristics to consider a set of signs and symptoms as a psychiatric disease.

Although in the GDT the smartphone dependents presented the same changes in the SCR as in the IGT, there was no impairment in the GDT performance in these individuals. This finding can be explained by the fact that cold executive functions can contribute to GDT performance, independently of emotional feedback processing (32, 38, 39). In other words, when smartphone dependents have doubts about the probability of winning or losing, biased implicit emotional processing can influence the choice of disadvantageous alternatives, but when they know exactly the likelihood of winning or losing, explicit knowledge influences the decision of advantageous options. These results were similar to those found in one study with pathological buyers (44), and in two studies with pathological gamblers (45, 46). In these studies, individuals of case group presented a worse performance in the IGT when compared to controls, but there was no difference in the performance in the GDT between groups (44–46). As postulated by Damasio, impairment in decision-making under ambiguity is probably more detrimental to real life than impairment in decision making under risk (39).

Three studies found impairment in decision-making under risk in smartphone dependents (51–53). However, they used the Intertemporal Choice Test (ICT) to evaluate decision making. In the ICT individuals have to react to changing winning probabilities, while the probabilities in GDT are stable, allowing the establishment of long-term strategies (38). Therefore, the tests may measure different aspects of executive functions and, consequently, different aspects of decision-making under risk.

In the analysis of decision-making process in Internet dependents, most of the studies demonstrated that these individuals do not show impairment in the performance in the IGT (36, 37, 70). In addition, during the IGT performance, Nikolaidou et al. (37), demonstrated that Internet dependents had higher SCR after punishments and lower SCR after rewards, indicating a profile of hypersensitivity to punishments and hyposensitivity to rewards in these individuals. This profile is just the opposite of that presented by smartphone dependents. Therefore, the neuropsychological profile of Internet dependents may be different from that of smartphone dependents. Internet dependents (who use Internet preferably for online games on desktop computers) (71–73) are more introverted, have more social phobia, and are more sensitive to punishments (74–76). Therefore, they can use the Internet as a way to escape from punitive reality, as if they wanted to “escape from real life” and from social exposure. On the other hand, smartphone dependents (who use smartphone preferably for social networks engagement) (77, 78) are more extroverted, more impulsive and more likely to novelty-seeking (12, 15, 16, 76, 79–82). Therefore, they can use the smartphone as a way to broaden social contacts, seek new sensations and receive rewards. This difference in the profile may be another evidence to distinguish the constructs “Internet Addiction” and “Smartphone Addiction.”

The decision-making profile of smartphone dependents may reflect a dysfunction of the ventromedial pre-frontal cortex (VMPFC), and limbic system, with preserved functionality of the dorsolateral pre-frontal cortex (DLPFC) (83). More specifically, alterations in the functioning of the amygdala and other structures of the limbic system may cause less sensitivity to punishment and greater sensitivity to rewards (primary emotions). Some authors have suggested that, in substance addictions, there is initially a functional impairment of the VMPFC and the limbic system that favors the beginning and continuation of psychoactive substances use, even in face of possible future damages. On the other hand, the DLPFC impairment would occur lately in chemical addictions and would be caused by the direct neurotoxic effect of the drugs in this region. Therefore, it is plausible that in SA there is only impairment in decision making under ambiguity with preservation of decision under risk, since there is no direct effect of a chemical substance in the central nervous system (84–86).

The impairment in decision-making under ambiguity in smartphone dependents can be analyzed through the triadic model of decision-making (87). The impulsive system, represented mainly by the amygdala-striatum, contributes to automatic behaviors and habits (87). Its hyperactivation in SA may contribute to the compulsive ritual of regular checking of smartphones due to hypersensitivity to rewards (e.g., likes and comments on social networks) and hyposensitivity to punishments (e.g., traffic accidents, relationships problems). In addition, the influence of external stimuli (such as smartphone vibration, emission of sounds and lights, or even the visualization of people using the device) can contribute to the hyperactivation of the impulsive system, contributing to increase the smartphone use behavior. The reflexive system, represented mainly by the VMPFC, contributes to self-regulation and prediction of future consequences of the behavior (87). Its hypoactivation in SA may contribute to the difficulty to control the intensity and frequency of smartphone use, and to the difficult in recognizing its long-term disadvantages, such as academic and labor impairments. Finally, the insula system, which detects homeostatic perturbations translating internal signals into feelings of craving, increases the activation of the impulsive system and reduces the activation of the reflexive system. In SA, the hyperactivation of the insula may contribute to the identification of insight feelings and thoughts that trigger the search for the smartphone, increases impulsivity and reduces self-control. Therefore, the possible hyperactivation of the impulsive and insula system and the hypoactivation of the reflexive system in SA can impair the decision making under ambiguity, favoring abusive use of smartphones even in face of negative consequences or possibility of future negative consequences caused by this behavior. However, these assumptions are hypothetical, since the correlation between the impairment in decision-making and the functional alterations in brain circuits in SA can only be established by studies with functional imaging.

Decision-making under ambiguity reflects more strongly the reality of daily decisions, since most decisions in real life are made without the prior certainty of the probability of each outcome (39). In addition, it has already been shown that impairment in decision-making under ambiguity is more detrimental to daily life than impairment in decision-making under risk (39). Therefore, the functional decline in smartphone dependents daily lives can be consequent of the impairment in decision-making under ambiguity. The current findings have clinical implications as preventive strategies can focus on the development of emotional regulation, awareness of bodily signs / symptoms, and postponement of rewards. It has already been shown that these strategies can be achieved by physical exercises, focus attention training, mindfulness, biofeedback, interoceptive exposure therapy, and Cognitive-Behavioral Therapy (CBT) (87).

Our findings should be regarded considering some limitations. This study has a cross-sectional design and do not allow the establishment of cause and effect relationships. In addition, the only physiological parameter measured was skin conductance, which may have reduced the sensitivity and specificity of these measures. The use of fake money during decision-making tests may have induced a less cautious behavior of the individuals who participated in the study. Finally, the use of a sample without psychiatric comorbidities in the experimental group does not reflect the clinical presentation of the majority of the subjects with smartphone dependents, according to previous studies. However, we preferred to exclude subjects with psychiatric disorders from the experimental group to avoid the effects of mood in the performance in the decision-making process. Therefore, the creation of a homogenous experimental group made possible the evaluation of the exclusive influence of the dependence of smartphone on the modification of the decision-making process.

Therefore, we can assume that technology is not only a source of benefits. Our results suggest that some technologies, as smartphone use, may trigger patterns of mental dysfunctions in vulnerable subjects, similar to additive disorders. SA seems to be the same disease with another face, the dimensional syndrome of addiction that share biological and cognitive vulnerabilities. More studies are warranted to assess the cognitive dysfunction in a longitudinal design using neuroimaging approach in SA.

## Ethics Statement

All participants were informed about the voluntary nature of the study and its implications, and signed the Informed Consent Term. The study was submitted and approved by the Research Ethics Committee of the Federal University of Minas Gerais (UFMG) with the number C.A.A.E. number 54066516.0.0000.5149 and carried out according to the latest version of the Declaration of Helsinki.

## Author Contributions

All authors participated in the whole study process in an egalitarian way. All authors had full access to all data in the study and take responsibility for the integrity of the data and the accuracy of the data analysis.

### Conflict of Interest Statement

The authors declare that the research was conducted in the absence of any commercial or financial relationships that could be construed as a potential conflict of interest.
