# Corrigendum: Bad Choices Make Good Stories: The Impaired Decision-Making Process and Skin Conductance Response in Subjects With Smartphone Addiction

**DOI:** 10.3389/fpsyt.2019.00397

**Published:** 2019-07-01

**Authors:** Julia Machado Khoury, Luiz Filipe Silva Codorino Couto, Douglas de Almeida Santos, Vitor Hugo de Oliveira e Silva, João Pedro Sousa Drumond, Letícia Lopes de Carvalho e Silva, Leandro Malloy-Diniz, Maicon Rodrigues Albuquerque, Maila de Castro Lourenço das Neves, Frederico Duarte Garcia

**Affiliations:** ^1^Department of Mental Health, Universidade Federal de Minas Gerais–UFMG (Federal University of Minas Gerais), Belo Horizonte, Brazil; ^2^Department of Clinical Medicine, Faculty of Health and Human Ecology, Belo Horizonte, Brazil; ^3^Post-Graduation Program in Molecular Medicine (Pós-Graduação em Medicina Molecular), School of Medicine, Universidade Federal de Minas Gerais–UFMG (Federal University of Minas Gerais), Belo Horizonte, Brazil; ^4^Department of Sports, Universidade Federal de Minas Gerais–UFMG (Federal University of Minas Gerais), Belo Horizonte, Brazil; ^5^INCT of Molecular Medicine, Universidade Federal de Minas Gerais–UFMG (Federal University of Minas Gerais), Belo Horizonte, Brazil; ^6^Unité Inserm U1073, Rouen, France

**Keywords:** decision-making, game of dice task, Iowa gambling test, skin conductance, smartphone addiction, somatic markers

In the published article, there was an error in affiliations 1, 3, 4, and 5.

As required by our institution, “UFMG” should appear in the author affiliations. Instead of the following: “^1^Department of Mental Health, Federal University of Minas Gerais, Belo Horizonte, Brazil,” “^3^Post-Graduation Program in Molecular Medicine, School of Medicine, Federal University of Minas Gerais, Belo Horizonte, Brazil,” “^4^Department of Sports, Federal University of Minas Gerais, Belo Horizonte, Brazil,” and “^5^INCT of Molecular Medicine, Federal University of Minas Gerais, Belo Horizonte, Brazil” the affiliations should read as follows: “^1^Department of Mental Health, Universidade Federal de Minas Gerais–UFMG (Federal University of Minas Gerais), Belo Horizonte, Brazil,” “^3^Post-Graduation Program in Molecular Medicine (Pós-Graduação em Medicina Molecular), School of Medicine, Universidade Federal de Minas Gerais–UFMG (Federal University of Minas Gerais), Belo Horizonte, Brazil,” “^4^Department of Sports, Universidade Federal de Minas Gerais–UFMG (Federal University of Minas Gerais), Belo Horizonte, Brazil,” and “^5^INCT of Molecular Medicine, Universidade Federal de Minas Gerais–UFMG (Federal University of Minas Gerais), Belo Horizonte, Brazil.”

The authors declare that the research was conducted in the absence of any commercial or financial relationships that could be construed as a potential conflict of interest.

