# Supplementary material for: Bad Choices Make Good Stories: The Impaired Decision-Making Process and Skin Conductance Response in Subjects With Smartphone Addiction
Source: Front Psychiatry. 2019 Feb 22;10:73. doi: 10.3389/fpsyt.2019.00073 (PMC6395375; doi:10.3389/fpsyt.2019.00073)
Supplement: Supplementary file 1 [file Table_1.DOCX]

| **Authors** | **Year** | **Disorder** | **Decision under ambiguity** | **Decision under risk** | **Physiological parameters** |
| --- | --- | --- | --- | --- | --- |
| Cavedini et.al | 2002 | Pathological gambling (PG) | Individuals with PG had worse IGT performance when compared to controls | Not Evaluated | Not measured |
| Goudriaan et. al | 2005 | Pathological gambling (PG) | Individuals with PG had worse IGT performance when compared to controls | Not Evaluated | Not measured |
| Goudriaan et. al | 2006 | Pathological gambling (PG) | Individuals with PG had worse IGT performance when compared to controls | Not Evaluated | Controls had higher anticipatory SCR before disadvantageous choices, while individuals with PG presented no difference. There were no differences between the case and control groups with respect to the SCR after rewards and punishments. |
| Linnet et. al | 2011 | Pathological gambling (PG) | Individuals with PG had worse IGT performance when compared to controls | Not Evaluated | Individuals with PG had greater release of dopamine in the ventral striatum in PET |
| Lorains et. al | 2014 | Pathological gambling (PG) | Individuals with PG had worse performance in IGT than controls, and individuals with PG of non-strategic games had worse performance than those of strategic games | Not Evaluated | Not measured |
| Yan et. al | 2014 | Pathological gambling (PG) | Individuals with PG had worse IGT performance when compared to controls | Not Evaluated | Not measured |
| Fuentes et. al | 2014 | Pathological gambling (PG) | Individuals with PG showed improvement in IGT performance after drug treatment and CBT | Not Evaluated | Not measured |
| Brand et. al | 2005 | Pathological gambling (PG) | Not Evaluated | Individuals with PG had worse performance in GDT when compared to controls | Not measured |
| Labudda et. al | 2007 | Pathological gambling (PG) | Not Evaluated | Individuals with PG had worse performance in GDT when compared to controls | There was no difference in the level of salivary cortisol and salivary alpha-amylase during the GDT between groups. |
| Trotzke et. al | 2015 | Pathological buying (PB) | Individuals with BP had worse IGT performance when compared to controls | Individuals with PB had worse performance in GDT when compared to controls | Individuals with PB had lower SCR before disadvantageous choices than before advantageous choices in IGT. The physiological parameters were not altered during the GDT. |
| Sun e colaboradores | 2009 | Internet Addiction (IA) | Individuals with IA had worse IGT performance when compared to controls | Not Evaluated | Not measured |
| Si-Hua et. al | 2014 | Internet Addiction (IA) | Individuals with IA had worse IGT performance when compared to controls | Not Evaluated | Not measured |
| Ko et. al | 2010 | Internet Addiction (IA) | Individuals with IA had better IGT performance when compared to controls | Not Evaluated | Not measured |
| Metcalf et. al | 2014 | Internet Addiction (IA) | Individuals with IA had better IGT performance when compared to controls | Not Evaluated | Not measured |
| Nikolaidau et. al | 2016 | Internet Addiction (IA) | Individuals with IA had better IGT performance when compared to controls | Not Evaluated | Individuals with IA had higher SCR after punishment than after rewards |
| Pawlikowski et. al | 2011 | Internet Addiction (IA) | Not Evaluated | Individuals with IA had worse performance in GDT when compared to controls | Not measured |
| Yao et. al | 2014 | Internet Addiction (IA) | Not Evaluated | Individuals with IA had worse performance in GDT when compared to controls | Not measured |
| Hadar et. al | 2015 | Smartphone Addiction (SA) | Not Evaluated | Individuals with SA had worse performance in the Intertemporal Choice Test when compared to controls | Individuals with SA had less activation of DLPFC in fMRI |
| Wilmer and Chein | 2016 | Smartphone Addiction (SA) | Not Evaluated | Individuals with AS had worse performance in the Intertemporal Choice Test when compared to controls | Not measured |
| Tang et. al | 2017 | Smartphone Addiction (SA) | Not Evaluated | Individuals with AS had worse performance in the Intertemporal Choice Test when compared to controls | Not measured |
